# Identification of Alternative Splicing in Proteomes of Human Melanoma Cell Lines without RNA Sequencing Data

**DOI:** 10.3390/ijms24032466

**Published:** 2023-01-27

**Authors:** Anna A. Lobas, Elizaveta M. Solovyeva, Lev I. Levitsky, Anton O. Goncharov, Elena Y. Lyssuk, Sergey S. Larin, Sergei A. Moshkovskii, Mikhail V. Gorshkov

**Affiliations:** 1 V.L. Talrose Institute for Energy Problems of Chemical Physics, N.N. Semenov Federal Research Center for Chemical Physics, Russian Academy of Sciences, 119334 Moscow, Russia; 2 Federal Research and Clinical Center of Physical-Chemical Medicine, 119435 Moscow, Russia; 3 Dmitry Rogachev National Medical Research Center of Pediatric Hematology, Oncology and Immunology, 117198 Moscow, Russia; 4 Faculty of Biomedicine, Pirogov Russian National Research Medical University, 117997 Moscow, Russia

**Keywords:** proteogenomics, cell lines, alternative splicing, melanoma, proteomics

## Abstract

Alternative splicing is one of the main regulation pathways in living cells beyond simple changes in the level of protein expression. Most of the approaches proposed in proteomics for the identification of specific splicing isoforms require a preliminary deep transcriptomic analysis of the sample under study, which is not always available, especially in the case of the re-analysis of previously acquired data. Herein, we developed new algorithms for the identification and validation of protein splice isoforms in proteomic data in the absence of RNA sequencing of the samples under study. The bioinformatic approaches were tested on the results of proteome analysis of human melanoma cell lines, obtained earlier by high-resolution liquid chromatography and mass spectrometry (LC-MS). A search for alternative splicing events for each of the cell lines studied was performed against the database generated from all known transcripts (RefSeq) and the one composed of peptide sequences, which included all biologically possible combinations of exons. The identifications were filtered using the prediction of both retention times and relative intensities of fragment ions in the corresponding mass spectra. The fragmentation mass spectra corresponding to the discovered alternative splicing events were additionally examined for artifacts. Selected splicing events were further validated at the mRNA level by quantitative PCR.

## 1. Introduction

More than 90% of multiexon human genes undergo alternative splicing [1,2]. Moreover, most of the genes give rise to more than two isoforms, and the current annotation of the human transcriptome contains an average of four distinct products per gene [3]. Thus, alternative splicing drastically expands the diversity and complexity of gene products (mRNAs) to allow tissue and organ specificity [2,4]. This diversity could be one of the reasons for mammalian complexity and play a crucial role in development and evolution [5]. Bound to its important role in cell biology, dysregulation of alternative splicing often leads to complicated pathological conditions.

To date, a number of diseases, including different types of muscle atrophies and cardiomyopathy, neurodegenerative diseases, and even aging [6], have been associated with a change in the frequency of exon/intron inclusion (for a review, see [7,8]). Such alterations can lead to changes in the translation process, as well as to the production of proteins with different functions (or non-functional ones). Splicing dysregulation was repeatedly reported in cancer cells, directly affecting cancer-related genes, as well genes important for splicing process (splice factors and spliceosomal subunits) [9]. Moreover, a number of those mis-spliced transcripts and their encoded proteins were shown to drive cancer progression or contribute to various cancer hallmarks [10]. Melanoma is an aggressive skin cancer currently considered one of the most dangerous human malignant tumors. In recent years, alternative splicing dysregulation has been shown to influence the pathogenesis of melanoma [11,12,13,14,15,16,17,18,19,20] and is associated with the prognosis of patient survival [18]. Despite the undoubted importance of alternative splicing regulation and the crucial role of protein products from mis-spliced mRNAs, nowadays most of the studies are still conducted on the transcript, rather than protein level.

The presence of numerous alternative transcript isoforms was repeatedly shown in various tissues, organisms, and disease contexts [2,21,22,23,24,25,26]. However, the existence of protein isoforms, e.g., the ones produced from alternatively spliced transcripts, is still an actively debated question [10,27,28,29,30,31]. A number of earlier proteomic studies have demonstrated the presence of multiple protein isoforms [32,33], supported further by mass spectrometry-based analyses of proteomes of large varieties of tissues and organs [34]. However, recent studies and re-analysis of the previously reported findings showed a surprisingly low level of protein isoforms in normal human tissues [28,35], suggesting that the nervous and muscle tissues express the highest (yet still low) number of tissue-specific protein isoforms [36]. There are a number of reasons for such dichotomy in mass spectrometry-based bottom-up proteomics, including (i) low protein sequence coverage translating into a low probability to detect splicing-specific peptides, (ii) a statistically increased probability of arginine or lysine being encoded at exon boundaries [37], (iii) sample-specific alternative splicing, and (iv) false discovery rate estimation and false positive identifications. The first challenge can potentially be overcome by using targeted techniques, such as MRM/PRM [38], assuming there is prior knowledge about proteins of interest or, to some extent, data-independent acquisition (DIA) methods [27]. The latter is bound to the almost exclusive use of trypsin, which cleaves sequences specifically after arginine and lysine residues and, therefore, lowers the probability of producing peptides covering the splice junction. A number of alternative proteases can be used to solve this problem [39]. Then, false identifications are an intrinsic feature of the target-decoy approach (TDA) commonly used in proteomics [40]. Indeed, the TDA-based filtering implies a particular percentage (usually 1%) of false-positive identifications, which can be neglected in the proteome-wide analysis. However, when a particularly small subset of peptides is considered (e.g., peptides corresponding to non-canonical isoforms), the percentage of false positives can reach 100%. Therefore, group-specific filtering is needed to come up with reliable identification of splicing-related peptides, which was the topic of recent intensive discussion in the literature [28,41,42,43]. The problem can be addressed by using sample-specific databases, although it would require a parallel RNAseq analysis of all expressed transcripts and isoforms for the same samples. This approach has many benefits, allowing the detection of novel protein isoforms produced from mis-spliced sample-specific transcripts [38]. However, apart from the cost of analyses for large sample cohorts, it cannot be used for the re-analyses of existing proteomic datasets, samples available in limited amounts, as well as physiological liquids. 

In this work, we developed an approach for the identification of sample-specific splicing events at the proteome level without *a priori* transcriptional information. The efficiency of the approach was evaluated by re-analyzing previously published proteomics datasets acquired for melanoma samples [44]. First, we evaluated different properties of peptide identifications, such as retention time, MS/MS spectra, among others, and estimated their utility for the filtering of identifications corresponding to known non-canonical isoforms. Then, the developed filtering was integrated with a proposed method of transcriptome-free identification of novel protein isoforms based on the combinatorial pairing of known exons.

## 2. Results

LC-MS/MS proteomic datasets obtained earlier for four melanoma cell lines, KIS, KOR, P, and 82 from the previous study [44] were used to develop the method and test its efficiency. These cell lines were selected based on the depth of respective proteome coverage and reproducibility between the biological replicates. On average, about 17,500 peptides and 3500 proteins have been identified for each of the cell lines. Such sensitivity makes it possible to count on the detection of splicing-specific peptides by classic “bottom-up” proteomics methods without additional enrichment.

### 2.1. RefSeq-Based Identification

The first approach to the identification and validation of alternative splicing products without having the sample-specific RNA sequencing data at hand was the use of a database of all previously detected transcripts. To evaluate this approach, we used the RefSeq human protein database (Genome-Build. GRCh38. https://www.ncbi.nlm.nih.gov/refseq/ (accessed on 14 May 2021); p. 13) containing 81,565 unique protein sequences, which is approx. four times the number of unique sequences (20,324) in the SwissProt canonical protein database.

**Figure 1:**
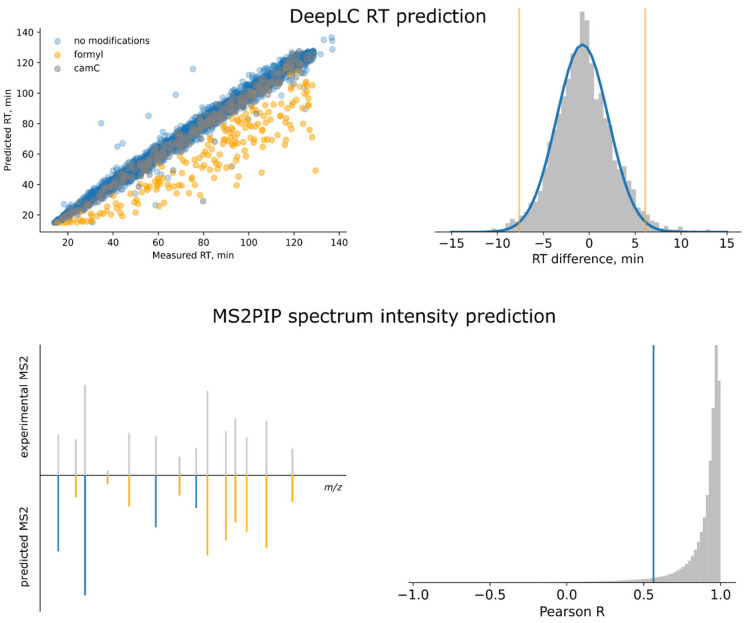
Two-step filtering of splicing peptide identifications was implemented as follows. DeepLC retention time prediction model calibration using a randomly selected half of all peptide identifications; LC filtering based on a 2-sigma threshold; and Pearson correlation filtering based on the prediction of the peptide ion fragmentation pattern using the MS2PIP tool [45]. The threshold value for MS2PIP filtering was selected at the 5th percentile of the corresponding distribution.

For the studied cell lines, 252 (line KIS) to 348 (line P) unique peptides not corresponding to canonical protein isoforms were identified. In order to increase the reliability of these peptide identifications, we have developed the following filters (Figure 1):

1. Chromatographic (LC) filter: Deviation of experimentally measured retention time for an identified peptide from the predicted one;
2. Fragmentation pattern filter: A Pearson correlation between fragment ion intensities in measured tandem mass spectra and predicted fragmentation pattern for a peptide ion in question.

Peptide retention time directly depends on the amino acid sequence and thus can be used as an additional confirmation of identifications [46]. Retention times were predicted using a recently introduced machine learning-based algorithm DeepLC [47]. To determine the LC filtering thresholds for the identification results, the prediction model was calibrated using one-half of the identified peptides (randomly selected) for the particular analysis. Figure 1 shows the correlation between predicted and experimental retention times for one of the LC-MS/MS runs. On average, the prediction accuracy of the DeepLC model was reasonably high, with a standard deviation of 3.7 to 4.1 min between experimental and predicted RTs for 140 min gradients. However, we systematically found that the predicted times differ significantly from the experimental ones for peptides with a modified N-terminus and, therefore, we excluded these peptides from the LC filtering step. For all other peptides, the distribution of prediction errors was calculated and approximated by a normal distribution (Figure 1). All identified peptides with a prediction error of more than 2 standard deviations (or *z*-scores above 2) were considered false matches and filtered out.

Relative intensities of peptide fragments in tandem mass spectra (MS/MS) depend on several parameters, such as peptide bond strength, neighboring amino acids, gas-phase basicities of the corresponding oxonium ions, fragment size, etc. These parameters together make the fragmentation pattern sequence-specific and, thus, the fragment ion intensities can be used to validate identifications. In this work, we employ the MS2PIP tool based on machine learning to predict fragment ion intensities for the identified spectra, [45] and develop a filtering procedure utilizing the deviation between experimental and predicted fragmentation patterns. In particular, for each identified peptide, the mass spectrum with the highest hyperscore assigned by the proteomic search engine was selected; this spectrum was then compared with the theoretical one and the Pearson correlation between these spectra was calculated for predicted fragments. Figure 1 shows the distribution of the obtained correlation *R* values for the peptides identified in one of the LC-MS/MS runs. The threshold value was set at the 5th percentile of this distribution.

**Figure 2:**
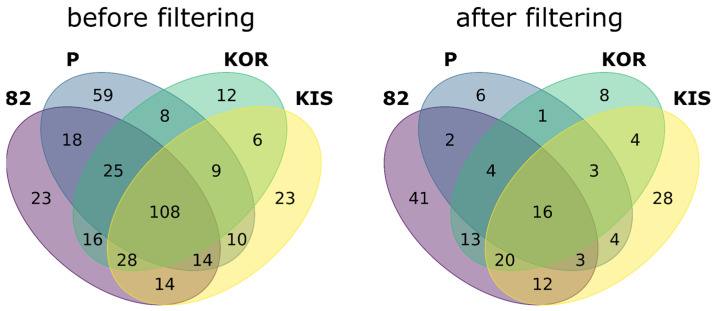
Numbers of peptides with alternative splicing were identified in four melanoma cell lines using the RefSeq database before and after the application of three-step filtering.

Furthermore, we considered only the peptides identified in all three replicates with the match between the runs setting of the search engine, meaning that the corresponding peptide-like feature was present in precursor (MS1) spectra. The use of the above filters resulted in a decrease in the number of uniquely identified peptides from the RefSeq database by 63–89%, depending on the cell line. Figure 2 shows the intersection of unique splicing-related peptides between cell lines before and after the use of the above filtering. The complete output of the searches and filtering is given in Supplementary Table S1.

### 2.2. Combinatorial Database Identification

A combinatorial database (CombiDB) was constructed based on RefSeq reference genome sequences and annotation (version GRCh38). The database was constructed as an annotated list of novel peptides, rather than whole artificial proteins, for the sake of computational performance. Each peptide would originate from a splicing event between two exons of the same gene, each represented in at least one transcript of that gene. The possible pairs of exons were constrained based on the following criteria:

1. The exons must not overlap;
2. If an exon contains an untranslated region (UTR), it cannot be coupled with another exon at the end where the UTR is located;
3. There must be no more than ten skipped (non-overlapping) exons between the two exons in the pair.

For each pair of exons, their coding parts were concatenated and translated, then tryptic peptides that span the junction and have a length between 7 and 50 amino acids (with up to one missed cleavage site) were added to the database.

This procedure produced 886,795 tryptic peptides that were not found in the in silico tryptic digest of the RefSeq protein database. Some of them, however, could still be found in RefSeq proteins as non-tryptic peptides; the exclusion of those left 885,359 novel peptides. For the purpose of this estimation, leucine and isoleucine were considered identical.

To validate the generation procedure, we assessed how many known junction-spanning peptides were generated. To this end, we repeated the same procedure but only considered pairs of consecutive exons from the same transcripts. This would produce peptides that are coded by annotated transcripts and must be present in the RefSeq protein database. We observed that of 449,708 peptides generated in this manner, 99.8% were present in the protein database. The remaining 0.2% indicates the discrepancies between the genome annotation and the protein database. On the other hand, 99.6% of RefSeq-derived peptides were present in the combinatorial database, indicating that the generation approach is comprehensive enough to cover most of the possible splicing events.

For the database search, we combined the tryptic digest of the whole RefSeq protein database with novel combinatorial peptides and generated decoys. For decoy generation, we reversed the peptide sequences, while keeping the N- and C-terminal residues in place. If the resulting peptide coincided with a target peptide or a previously generated decoy, the inner part of its sequence was shuffled (up to 20 attempts were made). Overall, the search database contained 1,591,886 RefSeq peptides, 885,359 novel peptides and their decoy versions. The total database size was 4,954,365 peptides: 2,477,245 targets and 2,477,120 decoys.

**Figure 3:**
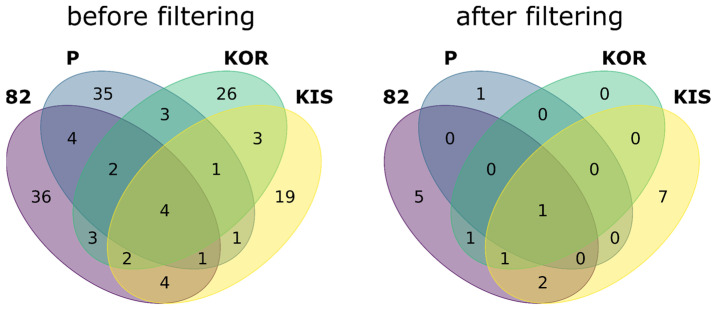
Numbers of novel AS events were identified at the protein level using the CombiDB before and after three-step filtering.

After the search against this database, all peptides corresponding to the RefSeq database were excluded from the search results, while the remaining combinatorial peptides corresponding to novel splicing were subject to the same three-step filtering procedure described above for the RefSeq identifications. The results of filtering are summarized in Figure 3; 68% to 94% of matches were filtered out depending on the cell line. Full search and filtering results are given in Supplementary Table S2, including the genomic coordinates of the novel junctions.

### 2.3. PCR Validation of Target Splicing Events

PCR validation of novel splicing events was performed in two cell lines with the highest quality proteomic data, 82, and KIS. Based on the search and filtering results, the following targets were selected for validation using real-time polymerase chain reaction (PCR): TLIINGLR peptide from *SMPD4*-encoded protein, KYADLLLK from *TXLNG*, LGILGLFQK from *NLGN4X*, and TWDQVPFSVSVSQLR from *PMEL*. We also checked these targets for artifacts using blastp. The peptide from *TXLNG* did not pass the blastp check since it might have been a misassigned peptide from a different gene, and the three remaining targets were considered for further validation.

**Figure 4:**
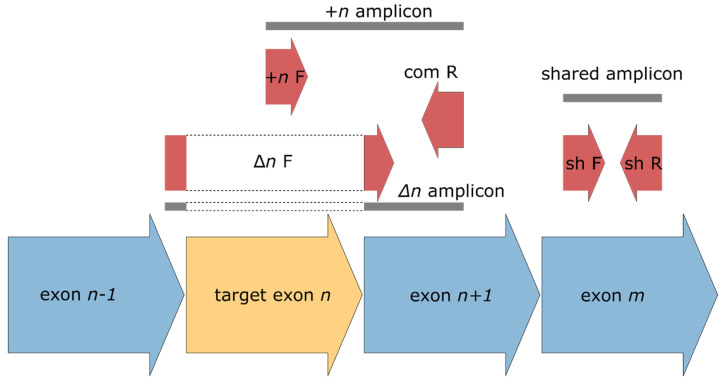
Schematic of primer selection for qPCR. Primers and the corresponding amplicons are shown in red and gray colors, respectively. Δn corresponds to an isoform with skipped target exon *n*, while +n means inclusion of the same exon. ‘com’ means the primer common for both isoform-specific amplicons, while the primers corresponding to the shared amplicon (used for intensity normalization) are designated as ‘sh’. F stands for forward primers and R for reversed ones.

The primer design criteria were as follows (Figure 4). For each target gene, one primer corresponding to exon skipping was selected so that it spans the junction (Δ*n*), while the alternative primer was selected from the skipped exon; the corresponding reversed primer was common for both isoforms. A pair of primers was also selected from the sequence region shared between all known isoforms in order to generate a shared amplicon for intensity normalization. Primer sequences are listed in Supplementary Table S3.

**Figure 5:**
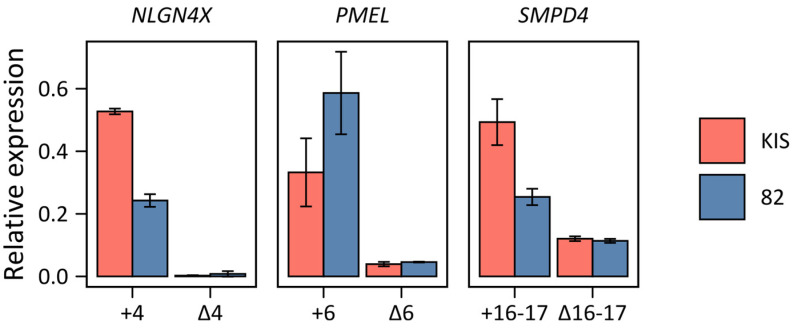
Relative mRNA expression of splice variants determined by qPCR. Bars indicate the average level of expression of the variant relative to the expression of the shared sequence; error bars represent standard deviation.

The results of real-time PCR analyses are shown in Figure 5. For the *NLGN4X* gene, the amount of amplicon with the skipped exon 4 was insignificant, while for the other two targets, the presence of both splice isoforms was confirmed in both cell lines. This observation suggests further that we deal with the novel splicing of these genes found at the proteome level.

## 3. Discussion

The size of the database used for a search in proteomics was discussed elsewhere [48]. It is trivial that you can only find what you have in the search database, yet the inflation of the search space by adding more and more protein sequences competing for the spectra may result in a decrease in sensitivity due to the increased number of false matches. While some researchers use a full protein database with isoforms, such as human UniProt for all searches, it may be suboptimal and further complicate the downstream analysis. Many choose to stick with the sequences present only in the SwissProt database containing “canonical” proteins; however, researchers may potentially lose the possible alternative splicing events. Herein, we suggest using the RefSeq human protein database (Genome-Build. GRCh38. https://www.ncbi.nlm.nih.gov/refseq/ (accessed on 14 May 2021), p. 13), in order to identify alternative splicing, containing 81,565 unique protein sequences, which is approx. four times the number of SwissProt sequences (20,324) (https://www.uniprot.org/proteomes/UP000005640 (accessed on 9 April 2021)). The intersection of the protein sequences contained in these databases is shown in Supplementary Figure S1a. Potentially, such an increase in the search space could lead to a decrease in the sensitivity of a search. However, since bottom-up proteomics deals with peptides rather than the proteins themselves, it is the size of the search space at the peptide level that matters (Supplementary Figure S1b). At this level, the real search space increases only by ~10%, which should not be a problem with sensitivity by all counts.

Supplementary Figure S2 shows the intersection between identified peptides using the two databases described above (only peptides identified in all three biological replicates). For all cell lines, searches against the RefSeq database resulted in the discovery of more peptides compared to searches against the canonical database, supporting the above conclusion that a marginal increase in the search space at the peptide level does not result in a loss of sensitivity. Therefore, databases containing proteins corresponding to all previously discovered transcripts can be effectively used for identification at the peptide level in bottom-up proteomics in spite of their large size. Moreover, since peptides found using only the RefSeq database (indicated as “unique” in Figure S2) correspond to alternative protein isoforms, such a comparative analysis should allow the detection of splice-specific peptides as well.

The combinatorial splicing database, on the other hand, introduces a more significant expansion to the database, adding 885,359 novel peptides, which corresponds to an approximately 50% increase in the database size compared to RefSeq. Here, we performed a study on how much this extension affects the identification of peptides corresponding to alternative splicing from the RefSeq database. Supplementary Figure S3 shows the numbers of RefSeq alternative splicing peptides identified in both RefSeq and combiDB searches (intersection) and exclusively in the RefSeq one (lost). This shows that a 50% expansion of the peptide sequence database leads to a loss of up to 10% of identified peptides, which is reasonable and does not undermine the approach based on a combinatorial database.

Having said that, we believe that using the combiDB gives insights into some novel alternative splicing. For instance, a peptide corresponding to novel splicing in melanocyte-specific transmembrane glycoprotein (*PMEL*, also known as gp100L) was identified in cell line 82, and the presence of the corresponding transcript was further confirmed by qPCR in both 82 and KIS cell lines. The annotated mass spectrum of the corresponding peptide and the mapping of this novel isoform in comparison with known isoforms of the *PMEL* gene are shown in Supplementary Figure S4. This protein is involved in amyloid formation and plays a critical role in the transition of melanosomes from stage I to stage II [49,50]. *PMEL* expression level was proposed to be a negative prognostic marker in Skin Cutaneous Melanoma (SKCM) [51]. Moreover, this protein has been used as a target antigen in some variants of adaptive T-cell therapy for melanoma [52]. The identified alternative splicing corresponds to a deletion of exon 5 (genomic coordinates chr12:55,957,921-55,958,085), which represents the main part of the amyloidogenic unit [53]. Such deletion would also affect two antigenic peptides (G9-154 and G9-209), which reportedly stimulate an anti-tumor immune response [54], and G9-154 (154-162 epitope) was used in the clinical trial of the T-cell therapy for metastatic melanoma [trial NCI-07-C-0174 and NCI-07-C-0175, www.ClinicalTrials.gov]. The identified novel splicing may play a crucial role in therapy, as well as in the early detection of melanoma. Further study is needed to assess the functional role of the identified isoform, as well as its presence in vivo. Thus, the proposed approach with proper validation can lead to the discovery of biologically significant novel alternative splicing, even in the absence of personalized transcriptome information.

It should be also noted that the studies on cell lines are just the first step toward the establishment of any kind of biomarker, while for clinical samples more issues may arise, for instance, from tumor heterogeneity [55,56]. Those issues have to be addressed at the corresponding stages of research.

## 4. Materials and Methods

The dataset of LC-MS/MS analyses of human melanoma cell lines on the Orbitrap Q Exactive mass spectrometer from an earlier study was used (available at ProteomeXchange with the dataset identifier PXD007662).

The searches for alternative splicing events at the proteome level were performed using FragPipe software based on the MSFragger search algorithm [57] with the match between runs option. This option was chosen to improve the reproducibility of biological replicate analyses. All results were filtered to a 1% false discovery rate.

The combinatorial database was constructed based on RefSeq reference genome sequences and annotations (version GRCh38) as an annotated list of novel peptides rather than whole artificial proteins, for reasons of computational performance. Each peptide would originate from a splicing event between two exons of the same gene, each represented in at least one transcript of that gene. Below is the pseudocode illustrating the database generation procedure:

- For each gene's exon set:

0. total # of exons := N.

1. for exon e_i_, i = 1…N−1.

- For j in 1…10.
- If the frame of exon e_j+i_ corresponds to the end of e_i_.
- Generate junction from exons e_i_ and e_i+j_.
- Apply the trypsin cleavage rule to the generated junction sequence and make peptides with up to one missed cleavage.
- Add junction-containing tryptic peptides with lengths of 7 to 50 amino acid residues to the peptide database.

For the database search, we combined the tryptic digest of the whole RefSeq protein database with novel combinatorial peptides and generated decoys. For decoy generation, we reversed the peptide sequences, while keeping the N- and C-terminal residues in place. If the resulting peptide coincided with a target peptide or a previously generated decoy, the inner part of its sequences was shuffled (up to 20 attempts were made).

All identified noncanonical peptides from RefSeq and combinatorial databases were validated using retention time and fragment intensity prediction by DeepLC [47] and MS2PIP [35] algorithms, respectively. Only peptides with non-zero intensity in all three replicates were considered.

Identified novel junction peptides selected as targets for PCR-based validation were additionally checked using BLAST to rule out other possible explanations for observing them. For each identified peptide, all its possible “isoforms” resulting from I/L replacement were generated and written into a FASTA file. Then, this file was searched using blastp (https://blast.ncbi.nlm.nih.gov/Blast.cgi (accessed on 1 April 2022)) against all human proteins. Some of the peptides identified in modified form had BLAST hits differing by a single amino acid and were deemed unreliable.

Primers were designed using Unipro UGENE [58] and Primer-BLAST [59]. Oligonucleotides were purchased from Evrogen (Evrogen, Moscow, Russia). All primers were checked for the formation of secondary structures, homo-, and hetero-dimers using the IDT OligoAnalyzer (http://scitools.idtdna.com/analyzer/Applications/OligoAnalyzer/ (accessed on 1 May 2022)). Primer sequences are presented in Supplementary Table S3.

Melanoma cells were cultivated the same way as in an earlier study [44]. Cell lines 82 and KIS were derived in 2005−2008 from excised tissues of stage IV metastatic malignant skin melanomas, as described elsewhere [60,61], and stored frozen in liquid nitrogen in the biobank of the Institute of Gene Biology, Russian Academy of Sciences. All cell lines were defrosted and cultured. The KIS cell line was cultured in the RPMI-1640 medium supplemented by 10% (*v*/*v*) fetal calf serum, 2 mM L-glutamine, 100 U/mL penicillin, and 100 mg/mL streptomycin. Cell line 82 was cultured in the DME/F-12 medium supplemented by 10% (*v*/*v*) fetal calf serum, 2 mM L-glutamine, 100 U/mL penicillin, 100 mg/mL streptomycin, and 15 mM HEPES. Cells were incubated at 37 °C and 5% (*v*/*v*) CO2, and the media were refreshed every 3 days. Those adherent cell lines were subcultured upon reaching 70−90% confluence. To this end, the medium was withdrawn from Petri dishes with cells. The dishes were washed with warm Dulbecco’s phosphate-buffered saline (PBS) with depleted Ca^2+^ and Mg^2+^; then, a 0.05% (*w*/*v*) trypsin solution containing 0.2 g/L EDTA was added. Dishes were incubated at 37 °C for 5−10 min until cells were detached from the plastic. Equal volumes of fresh media were then added to the dishes, media were resuspended, and cells were planted out in a 1:3 to 1:5 ratio. All reagents for cell culture were purchased from GE Healthcare Life Sciences (HyClone brand, Marlborough, MA, USA). For storage before analysis, detached cells were precipitated by centrifugation, washed three times with PBS, and stored at −80 °C. For the qPCR analysis, three independent batches of each cell line were grown, each containing at least 1 million cells.

RNA was isolated from 10⁶ cells using an RNeasy Mini Kit (Qiagen), according to the manufacturer’s instructions. Quantity and integrity of all used RNA stocks were controlled using a Qubit Fluorometer (Thermo Fisher Scientific) and non-denaturing agarose gel electrophoresis. Total RNA (1 µg) was reverse transcribed using MMLV Reverse transcriptase (Evrogen, Moscow, Russia) with random (dN)₁₀–primer.

Quantitative PCR of cDNA samples was performed at least in triplicate with a Bio-Rad CFX96 Touch Real-Time PCR Machine (Bio-Rad). In a total, a volume of 25 µL was added to the cDNA, 5 µL of 5x qPCRmix-HS SYBR Mastermix (Evrogen, Moscow, Russia), and 200 nM of each primer. For amplification, 40 cycles were performed with the following steps: denaturation at 95 °C for 20 s, annealing for 15 s, and elongation at 72 °C for 15 s. Different annealing temperatures were used for different genes: *PMEL* and *NLGN4X* at 57.5 °C, and *SMPD4* at 58.0 °C.

The identity of the amplicons in each sample was controlled by melting curve analysis. For melting curve analysis, stained products after the last amplification cycle were kept at 65 °C for 30 s and melted by raising the temperature by 0.5 °C per second up to 95 °C.

## 5. Conclusions

An approach for the discovery of novel alternative splicing events in proteomics data based on the combinatorial peptide database combiDB was proposed. Some of the results obtained by the approach have been validated by orthogonal methods, further advocating for the feasibility of its use for the search of novel splicing events at the proteome level in cases when the information about transcriptome is unavailable. Specifically, we found novel splicing in melanocyte-specific transmembrane glycoprotein (*PMEL*) in the melanoma cell lines, which was further confirmed by qPCR and may be crucial for cancer proliferation and, thus, be targeted by novel therapeutic approaches.

## Data Availability

Not applicable.

## Supplementary Materials

The following supporting information can be downloaded at https://www.mdpi.com/article/10.3390/ijms24032466/s1.
