# Supplementary material for: Identification of Alternative Splicing in Proteomes of Human Melanoma Cell Lines without RNA Sequencing Data
Source: Int J Mol Sci. 2023 Jan 27;24(3):2466. doi: 10.3390/ijms24032466 (PMC9916885; doi:10.3390/ijms24032466)
Supplement: Supplementary file 1 [file ijms-24-02466-s001.zip › ijms-2101161-supplementary/Supplementary.pdf]

Identification of Alternative Splicing in Proteomes of Human Melanoma Cell Lines without RNA Sequencing Data

Supplementary Information

Supplementary Figures 2

Supplementary Tables' Captions 4

Supplementary Figures

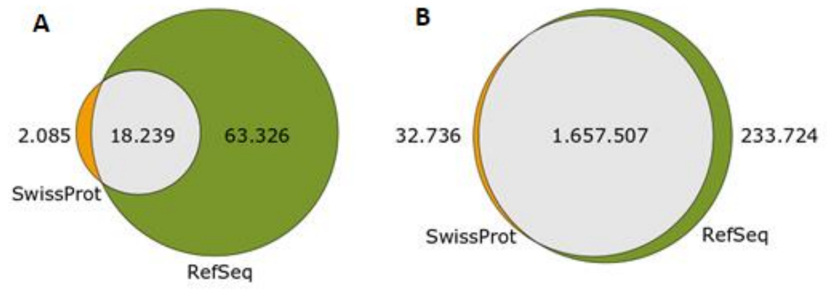

Figure S1. Intersection between SwissProt (containing only canonical sequences) and RefSeq (containing all known isoforms) databases at (A) protein and (B) peptide level

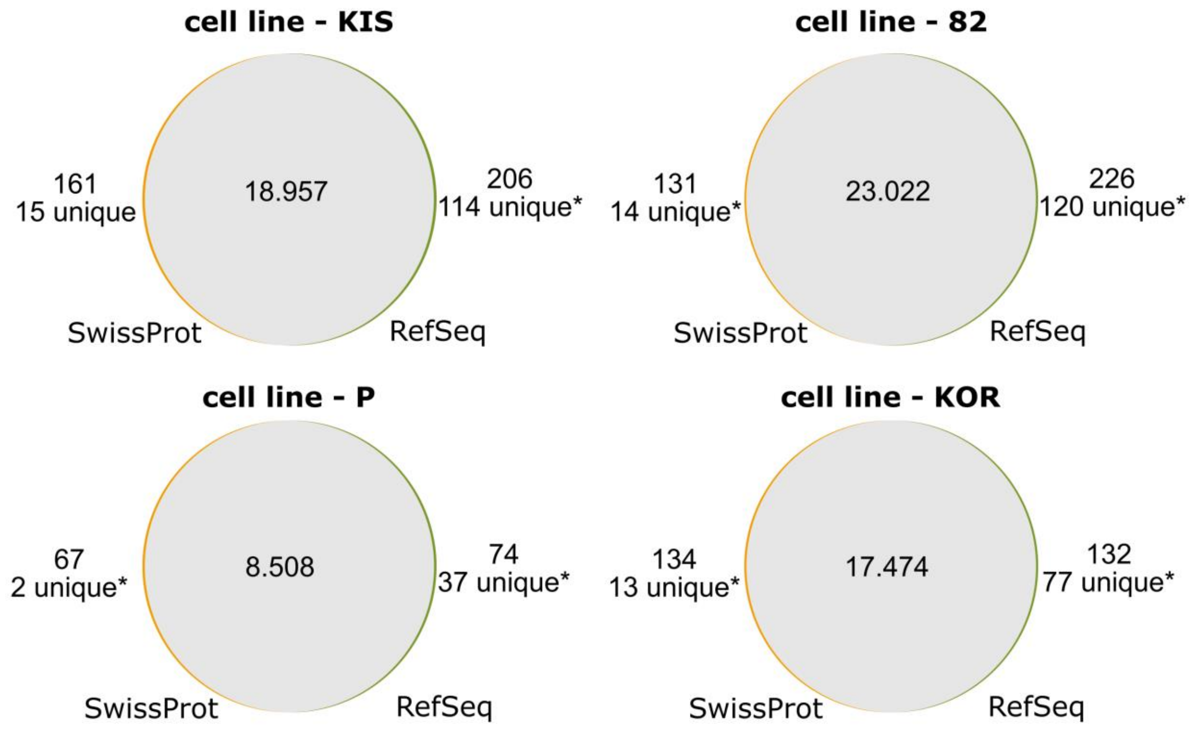

Figure S2. Comparison of the results of the searches against SwissProt and RefSeq sequence databases for four melanoma cell lines (KIS, 82, P, KOR). The numbers of peptides unique to the two databases is also shown next to the diagrams.

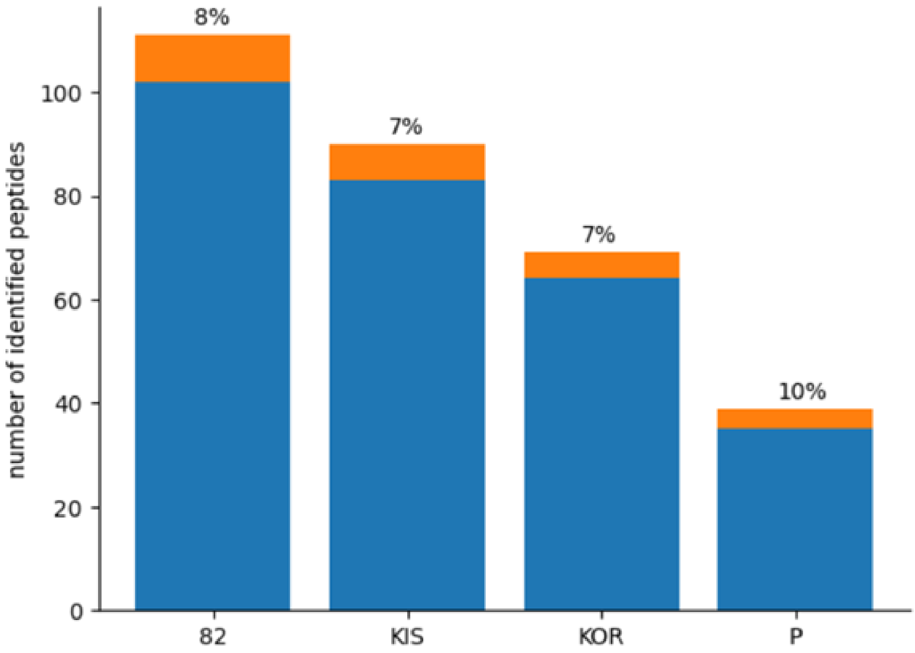

Figure S3. Number of RefSeq-specific peptides identified in the cell lines in both RefSeq and combiBD searches and exclusively in the RefSeq one.

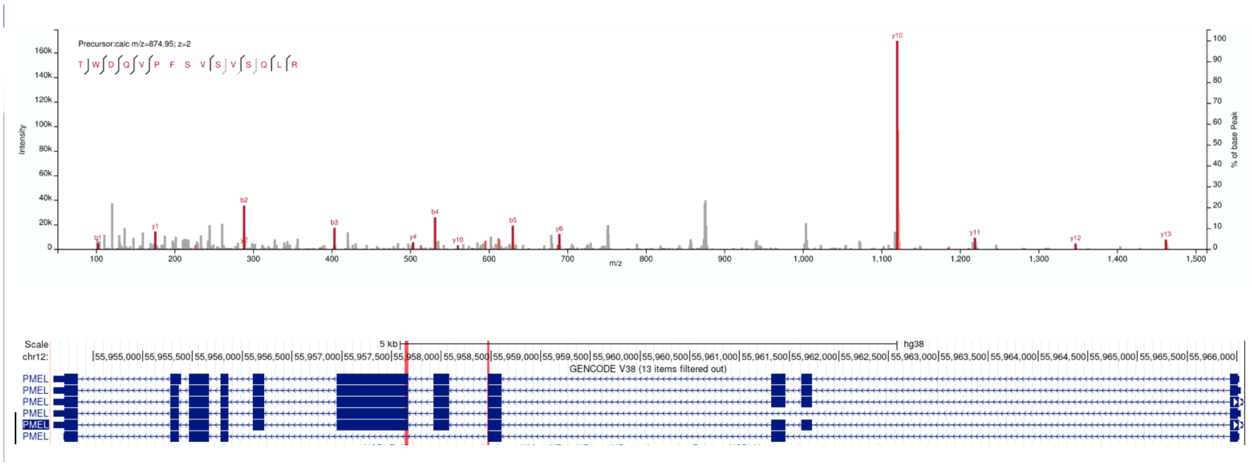

Figure S4. Novel splicing-specific peptide identified by use of combinatorial database TWDQVPFSVSVSQLR corresponding to PMEL gene. Top panel: fragmentation mass spectrum, matched peaks are shown in red. Bottom panel: genomic coordinates of the novel splicing event, the coordinates corresponding to the identified peptide are marked with red.

Supplementary Tables' Captions

Table S1. Identified peptides corresponding to alternative splicing from the RefSeq database. For each peptide and each cell line, the results of three-step filtering are given.

Table S2. Identified peptides corresponding to alternative splicing from the combinatorial database. For each peptide and each cell line, the results of three-step filtering are given, as well as the genomic coordinates of the corresponding novel splice junction.

Table S3. Primer sequences used for the real-time quantitative polymerase chain reaction.
